# Narrowing the Relationship between Human CCR5 Gene Polymorphisms and Chagas Disease: Systematic Review and Meta-Analysis

**DOI:** 10.3390/life13081677

**Published:** 2023-08-02

**Authors:** Jean Moisés Ferreira, Barbara Rayssa Correia dos Santos, Edilson Leite de Moura, Ana Caroline Melo dos Santos, Jean Carlos Vencioneck Dutra, Elaine Virgínia Martins de Sousa Figueiredo, José Luiz de Lima Filho

**Affiliations:** 1 Laboratório de Imunopatologia Keizo Asami—LIKA, Centro de Biocièncias, Universidade Federal de Pernambuco (UFPE), Recife 50670-901, Pernambuco, Brazil; 2 Secretaria de Estado de Educação do Espírito Santo (SEDU), Santa Lucia, Vitória 29056-085, Espírito Santo, Brazil; jeanvencioneck@gmail.com; 3 Laboratório de Biologia Molecular e Expressão Gênica—LABMEG, Departamento de Ciências Biológicas, Universidade Federal de Alagoas (UFAL), Campus Arapiraca, Arapiraca 57300-970, Alagoas, Brazil; brc.rayssa@gmail.com (B.R.C.d.S.); edilsonleite17@gmail.com (E.L.d.M.); anacaroline12305@gmail.com (A.C.M.d.S.); elaine.figueiredo@arapiraca.ufal.br (E.V.M.d.S.F.)

**Keywords:** American trypanosomiasis, SNPs, chemokines

## Abstract

Our aim was to carry out a qualitative and quantitative synthesis of the influence of CCR5 genetic variants on Chagas disease (CD) through a systematic review. A total of 1197 articles were analyzed, and eleven were included in the review. A meta-analysis was conducted along with principal component analyses (PCAs). The polymorphisms found were analyzed using the SNP2TFBS tool to identify possible variants that influence the interaction with gene binding sites. Eleven studied variants were identified: rs2856758, rs2734648, rs1799987, rs1799988, rs41469351, rs1800023, rs1800024, Δ32/rs333, rs3176763, rs3087253 and rs11575815. The studies analyzed were published between 2001 and 2019, conducted in Argentina, Brazil, Spain, Colombia and Venezuela, and included Argentine, Brazilian, Colombian, Peruvian and Venezuelan patients. Eight polymorphisms were subjected to the meta-analysis, of which six were associated with the development of the cardiac form of CD: rs1799987—G/G and G/A in the dominance model and G/G in the recessiveness model; rs2856758—A/G in the codominance model; rs2734648—T/T and T/G in the dominance model; rs1799988—T/T in both the codominance and recessiveness models; rs1800023—G allele and the G/G genotype in the codominance and recessiveness models, and the G/G and G/A genotypes in the dominance model; and rs1800024—T allele. The PCA analyses were able to indicate the relationships between the alleles and the genotypes of the polymorphisms. The SNP2TFBS tool identified rs1800023 as an influencer of the Spi1 transcription factor (*p* < 0.05). A correlation was established between the alleles associated with the cardiac form of CD in this review, members of the C haplotype of the gene (HHC–TGTG), and the cardiac form of CD.

## 1. Introduction

Chagas disease (CD)—known as American trypanosomiasis—is a neglected disease that affects seven million patients worldwide. About 12–20 thousand patients die every year and approximately 75 million people are exposed to CD, which mainly affects low-income populations. [1,2,3]. After infection by the etiologic agent (*Trypanosoma cruzi*), the patient enters the acute phase, followed by the chronic phase. The chronic phase can be asymptomatic (patients with an undetermined outcome), it can present symptoms related to cardiac complications or those associated with the digestive system, or a mixed form of cardiac and digestive system complications simultaneously [3].

Some investigations have associated the genetics of the patient with the infection and evolution of CD [4,5], indicating that polymorphisms in cytokines, chemokines and cellular receptor genes may contribute to the outcome of this disease, since these genes have central roles in activating and/or regulating the immune system [6]. CCR5 stands out among the cell receptor genes already analyzed. The CCR5 gene is located on chromosome 21 (3p21.31) and encodes the CC chemokine receptor 5 protein, or “CCR5”, which is expressed in T cells and integrates various immune processes [7]. Several variants of CCR5 have been associated with CD; however, some of these genetic variants have called attention to inconclusive or contradictory results in different studies [8,9,10,11,12,13], making it difficult to understand the real contributions of this gene to CD.

Gene expression and bioinformatics analyses continue to list CCR5 as a fundamental element in the development of CD [14,15,16]. In order to elucidate the relationship between CCR5 polymorphisms and CD, this study aimed to carry out a literature review on the CCR5 polymorphisms involved in CD, as well as to establish the correlations between these polymorphisms and CD through meta-analyses.

## 2. Materials and Methods

### 2.1. Search Strategies

This review has been registered on the PROSPERO platform (https://www.crd.york.ac.uk/PROSPERO/, March 2023). The searches were based on the PICOS strategy [17], where the participants (P) were patients infected with *T. cruzi*; intervention (I) was the identification of CCR5 gene polymorphisms in patients; comparisons (C) were made with any groups (asymptomatic or uninfected) or subgroups (chronic Chagas disease) of patients with well-defined profiles; the outcome (O) was the presence or absence of the polymorphism in patients; and the types of studies (S) were genetic association studies.

### 2.2. Term Selection, DeCS Analysis and String Construction

To choose the groups of words used in the database searches, the guides and protocols established by WHO and PAHO for the management of CD patients were primarily read [1,18], as well as expert articles and reviews [2,19]. After these readings, the sentences that referred to or directly described the *T. cruzi* infection were highlighted and the terms (1) “chagas disease”, (2) “Trypanosomiasis” and (3) “*Trypanosoma cruzi* infection” were listed. Using the Health Sciences Descriptors tool—DeCS, from the BVS portal (https://decs.bvsalud.org/, January 2022)—the general synonyms of each term were sought, whose returns were: (1) “chagasic disease”, (2) “American trypanosomiasis”, “Trypanosome parasite”, “Trypanosome infection”, “*Trypanosoma cruzi*”, “Trypanosomosis”, and (3) “*T. cruzi*”, “*Trypanosoma cruzi*” and “*cruzi*’’ (references codes C01.610.752.300.900.200, C01.920.625 and SP4.012.148.149). The same method was used for the term “Genetic polymorphism”, whose results were “Polymorphic gene”, “Single nucleotide polymorphism”, “Genetic variation”, “Allelic variation” and “SNP’’ (reference codes G05.365.795, G05.365.795.598, G05.365, G05.380 and SP4.102.118).

After finding all the synonyms of each descriptor, through the direct counting method, both commonly repeated words and those that appeared only once were highlighted. Applying Boolean operators, the highlighted terms were used with variations in the suffixes to form the search strings and their variations: “Chaga* disease” OR “Trypanosom*” OR “cruzi” AND “Polymorphi*” OR “Gene*” OR “Variation” OR “SNP”.

### 2.3. Data Base

The searches were carried out without time restrictions in the advanced mode of the databases, using the string mentioned above and its variations in the “title” field. The databases used were the CAPES Journal Portal, SciELO, Scopus, Web of Science, PubMed and ScienceDirect. The searches took place between January and February 2022. Only peer-reviewed articles written in English (when available, with the intention of improving the quality of returns) were used as filters for returns. Subsequently, the articles included in the qualitative synthesis had their references checked to include articles that were not detected during the database searches. Information about the manuscripts (number of repetitions of the manuscript detected the in searches, language used in the publication, type of text and type of study) were organized using LibreOffice Calc.

### 2.4. Inclusion and Exclusion Criteria

The following inclusion criteria were used: (1) original publications, (2) genetic association studies, and (3) electronically available articles. The following exclusion criteria were used: (1) duplicate studies, (2) studies using an animal model, (3) in vitro studies, (4) correlation studies between the genetics of the parasite and the patient, (5) genetic analysis studies of vectors, (6) studies that did not evaluate CCR5 genetic polymorphisms, (7) literature reviews, and (8) conference abstracts.

### 2.5. Selection of Publications and Quality Analysis

The publications were initially analyzed by title and abstract using the inclusion and exclusion criteria in the returns. Two independent reviewers (JMF and BRCS) reviewed all the returns; inconsistencies between reviewers were resolved by discussing the publication with a third reviewer (ACMS).

The analysis of the quality of the studies was performed after the selection of the studies. The quality analysis tool “Strengthening the Reporting of Genetic Association Studies (STREGA)” consists of a genetic study checklist, evaluating each part of the scientific article (title, abstract, introduction, methods, results, discussion, and other information), where a total of 22 points can be reached in the study. Using STREGA, studies were classified as low quality (<9), medium quality (9–16) and high quality (17–22).

### 2.6. Data Extraction

Data on the study design, year of publication, country of origin of the study (based on the first author), the studied population, sample size, information on the profile of the patients in the analyzed group, analyzed polymorphisms, main results, laboratory confirmation method for infection and confirmation method for polymorphism were extracted.

### 2.7. Meta-Analysis

Data from the selected articles were organized using Google Sheets. Global allele and genotype frequencies, using data from the included articles, were calculated using the same tool. Meta-analyses were performed on all polymorphisms available in the RevMan 5.4 software, where the strength of association was calculated using a 95% confidence interval and odds ratio (OR), with the generated value OR < 1 considered as a protective factor (trend) or risk reduction, while OR > 1 values were considered as a risk factor (trend), the results were considered significant when *p* ≤ 0.05. Study heterogeneity was assessed using the standard test (Q test and I-squared). When I^2^ ≥ 50% and *p* ≤ 0.05, indicating significant heterogeneity, probably caused by variations between studies, we chose to use the random analysis model (standardized by the software); for all other cases, the fixed analysis model was used [20]. All study designs (case–control, cohort and cross-sectional studies) were eligible for the meta-analysis [21].

The influences of the alleles (mutated vs. wild) and genotypes in different models of inheritance were evaluated: codominance (mutated homozygote vs. wild homozygote, or heterozygote vs. wild homozygote), recessiveness (mutated homozygote vs. heterozygous + wild homozygote) and dominance (mutated homozygote + heterozygote vs. wild-type homozygote). Funnel-plot graphs were inspected for possible publication bias.

### 2.8. SNP2TFBS

To identify single nucleotide polymorphisms (SNPs) that may affect transcription factor binding, we used the Mapping SNPs to Transcription Factor Binding Sites (SNP2TFBS) web interface (https://ccg.epfl.ch/snp2tfbs/, May 2022).

### 2.9. Patient Classification

Using the data available in the publications, patients were grouped into two main groups: healthy individuals without *T. cruzi* infection were termed as non-infected (nonCP), while patients infected with *T. cruzi* were generically termed as chronic patients with Chagas disease (CP). The “CP” group was subdivided according to the presence or absence of symptoms into two smaller groups: chronic asymptomatic patients in an undetermined form (UndCP) and chronic symptomatic patients (SympCP). Patients in the “SympCP” group were divided into three groups according to the clinical manifestation of the chronic form of CD: patients who presented any type of cardiac alteration/complications resulting from CD were grouped as cardiac patients (CardCP), while those with complications associated with the digestive system were grouped as digestive patients (DigCP). Patients who had both forms involving heart and digestive problems were grouped as mixed complications (CarDigCP). In our comparisons, the presence of someone involved in the CardCP or DigCp group did not exclude them from participating in the CarDigCP group, and vice versa.

### 2.10. Statistical Analysis

In order to evaluate the interaction between the sample data, for the results of the global comparisons (using all comparison studies) where there was significance, principal component analysis (PCA) was performed using the Past 4.10 software (https://www.nhm.uio.no/english/research/infrastructure/past/, May 2022). For significant comparisons found in the quantitative synthesis, sample power was calculated using G*Power 3.1.9.7 (χ^2^-Goodness-of-fit, Post hoc, α = 0.05) [22,23], to determine the representativeness of the results in the population, values >80% were considered significant.

Exploratory statistical analyses were performed to establish comparisons between genetic factors and the development of the cardiac form of CD. For this purpose, the possible correlations between haplotypes and their respective constituent alleles were verified using Pearson’s correlation. The χ2 test was used to compare the frequency of the HHC haplotype between groups, adopting a significance level of 5%.

## 3. Results

### 3.1. Characterization of the Studies

**Figure 1:**
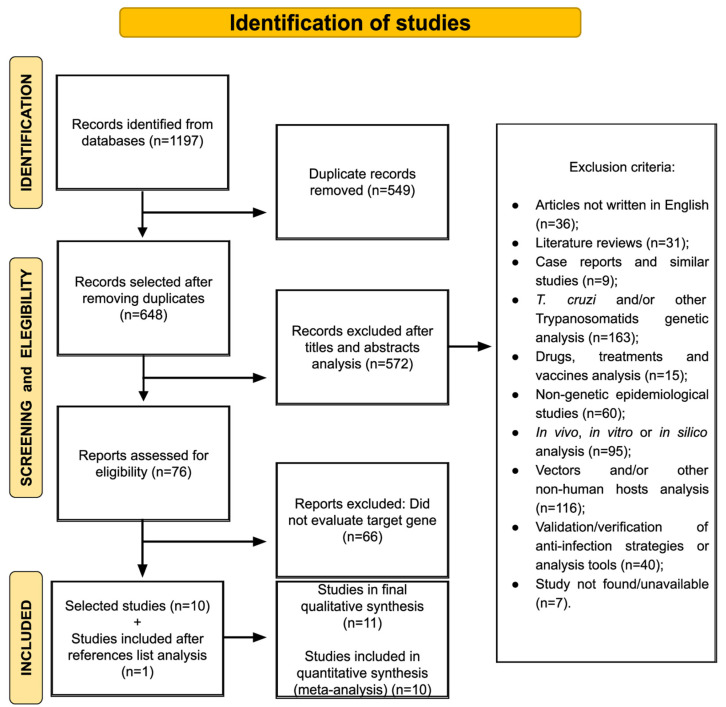
Flow of studies added in the systematic review. 1197 returns were collected from the databases, 11 studies were added in the qualitative synthesis, and 10 were used in the quantitative synthesis (meta-analysis).

Our work gathered the available information from the literature on the influence of CCR5 gene variants on CD. The flow of searches, analyses and the collection of articles is outlined in Figure 1. Table 1 summarizes the qualitative synthesis, with the characterization and main results of each of the eleven studies found in our search. One of the articles [24] proved to be a previous study of another with a larger sample [13]; therefore, we considered the most recent manuscript for the quantitative synthesis. Table 1 also presents the genetic variants used in the quantitative synthesis. The comparisons made between groups, the sample power found through G*Power, and the frequencies found are available in Supplementary Table S1.

### 3.2. Meta-Analysis and PCA

In our bibliographic survey, data on the genetic variants of CCR5 associated with CD were pooled, and eight genetic variants were subjected to a meta-analysis based on the number of studies: rs1799987, rs333, rs2856758, rs2734648, rs1799988, rs41469351, rs1800023 and rs1800024. The characteristics of the groups used in the studies that constituted the “CardCp” grouping are shown in Table 2. Only one study [13] analyzed patients with the digestive form of CD; therefore, comparisons were not possible using the “DigCP” grouping. During the analyses, two subgroups were formed based on the patients’ genetic backgrounds: patients from countries emancipated from Spanish colonization (with a predominantly Amerindian and European background) and Brazilian patients (with a predominantly Amerindian, European and African background).

#### 3.2.1. rs1799987

Eight studies evaluated the influence of the rs1799987 SNP on CD [8,9,10,12,13,26,27,28]. When comparing the groups of chronic vs. healthy controls (n = 558, CP = 304/nonCP = 254), symptomatic vs. asymptomatic (n = 1871, SympCP = 1031/UndCP = 840), healthy controls vs. asymptomatic (n = 370, nonCP = 254/UndCP = 116) and cardiac patients vs. asymptomatic (n = 1933, CardCP = 1093/UndCP = 840), no significant differences were found.

**Figure 2:**
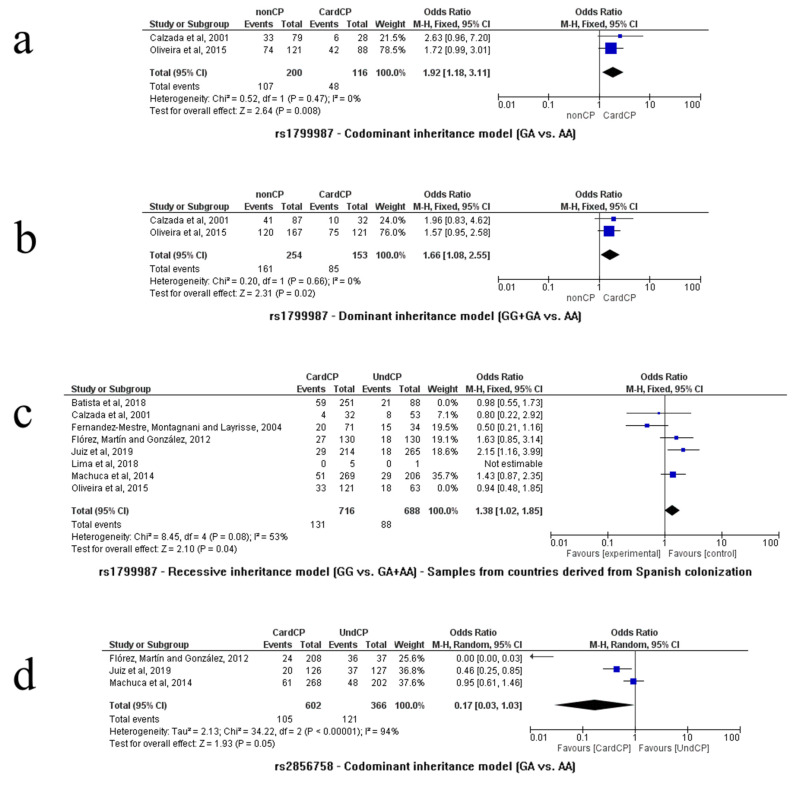
Meta-analysis of CCR5 genetic polymorphisms (rs1799987 and rs2856758) associated with CD. Comparisons were performed using dominance, codominance and recessiveness models. In our study, some analyses showed new associations based on investigated subgroups. The Mantel–Haenszel (M–H) test is the statistical method that was applied to generate an estimate of the association between Chagas disease and the analyzed risk factor after calculation adjustments [(**a**) codominant inheritance model GA vs. AA, (**b**) dominant inheritance model GG+GA vs. AA, (**c**) recessive inheritance model GG vs. GA+AA, and (**d**) codominant inheritance model GA vs. AA]. References: Batista et al., 2018 [8]; Calzada et al., 2001 [9]; Flórez, Martín and González, 2012 [10]; Fernandez-Mestre, Montagnani and Layrisse, 2004 [27]; Juiz et al., 2019 [26]; Lima et al., 2018 [12]; Machuca et al., 2014 [28]; and Oliveira et al., 2015 [13].

The comparison between patients in the healthy control group vs. cardiac patients (n = 407, nonCP = 254/CardCP = 153, [9,13]) showed, in the codominance model (A/G vs. A/A), an association of the heterozygous genotype with the risk of developing the cardiac form (*p* = 0.008, OR = 1.92, 95% CI (1.18–3.11), I^2^ = 0%, fixed model). Similarly, in the dominance model (A/G + G/G vs. A/A), an association of G carriers with the risk of developing the cardiac form was also verified (*p* = 0.02, OR = 1.66, 95% CI (1.08–2.55), I^2^ = 0%, fixed model). Both associations are summarized in Figure 2a,b, respectively.

**Figure 3:**
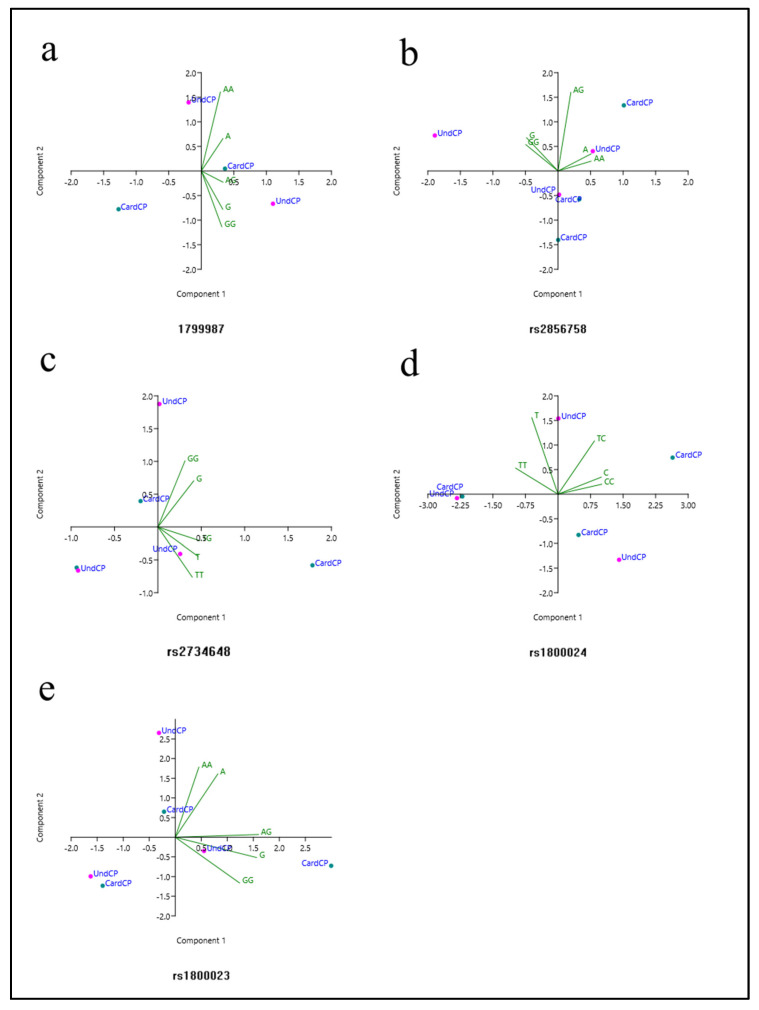
Principal component analysis (PCA) of genetic variants of CCR5 associated with CD. The established correlations were performed using the alleles and genotypes of each of the SNPs with statistical results on a global scale (without the need to analyze investigated subgroups). The green dots represent CD cardiac patient groups (CardCP), while the pink dots represent asymptomatic patients (UndCP). [(**a**) PCA between AA, AG and GG genotypes, and A and G alleles on SNP rs1799987; (**b**) PCA between AA, AG and GG genotypes, and A and G alleles on SNP rs2856758; (**c**) PCA between TT, TG and GG genotypes, and T and G alleles on SNP rs2734648; (**d**) PCA between TT, TC and CC genotypes, and T and C alleles on SNP rs1800024; and (**e**) PCA between AA, AG and GG genotypes, and A and G alleles on SNP rs1800023].

PCA was used to correlate the results obtained in the meta-analysis and establish inter-relationships between the variables, creating a smaller set of principal components (PCs). PC1 corresponded to 89.58% of the variance, while PC2 corresponded to 9.33%, totaling 98.91% of the variance. PC1 was dominated by the A and G alleles and the A/G genotype, while PC2 was predominantly dominated by the homozygous A/A and G/G genotypes. The analysis of the generated graphs suggested a correlation between the A allele and the A/A genotype, as well as suggesting a correlation between the G allele and the G/G genotype, while there was a distancing of the A/G genotype from these two groups (Figure 3a).

Although the global comparison between cardiac patients vs. asymptomatic patients showed no difference between the groups, when the analysis was limited to patients from countries originating from Spanish colonization, [9,10,26,27,28], n = 1404, CardCP = 716/UndCP = 688), in the recessiveness model (G/G vs. G/A + A/A), the G/G genotype was associated with the risk of developing the cardiac form (*p* = 0.04, OR = 1.38, 95% CI (1.02–1.85), I^2^ = 53%, fixed model), as shown in Figure 2c. Other statistical associations were not found.

#### 3.2.2. rs333

Four studies analyzed the Δ32 deletion [9,10,13,27] in the groups of cardiac patients vs. undetermined (n = 583, CardCP = 366/UndCP = 217), chronic patients vs. healthy controls (n = 951, CP = 692/nonCP = 259), healthy vs. cardiac patients (n = 625, nonCP = 259/CardCP = 366) and healthy controls vs. undetermined (n = 476, nonCP = 259/UndCP217); however, due to the rarity of the Δ32 allele in the populations studied, some comparisons could not be performed. In addition, for viable comparisons, no differences were found between the analyzed groups.

#### 3.2.3. rs2856758

The rs2856758 polymorphism was analyzed in three studies [10,26,28]. Only the comparison between cardiac patients vs. asymptomatic patients (n = 1255, CardCP = 654/UndCP = 601) was feasible. The codominance model (A/G vs. A/A) indicated an association between the heterozygous genotype and a decreased risk of developing the cardiac form of CD (*p* = 0.05, OR = 0.17, 95% CI (0.03–1.03), I^2^ = 94%, random model), as shown in Figure 2d. Other genetic models of association were not significant.

**Figure 4:**
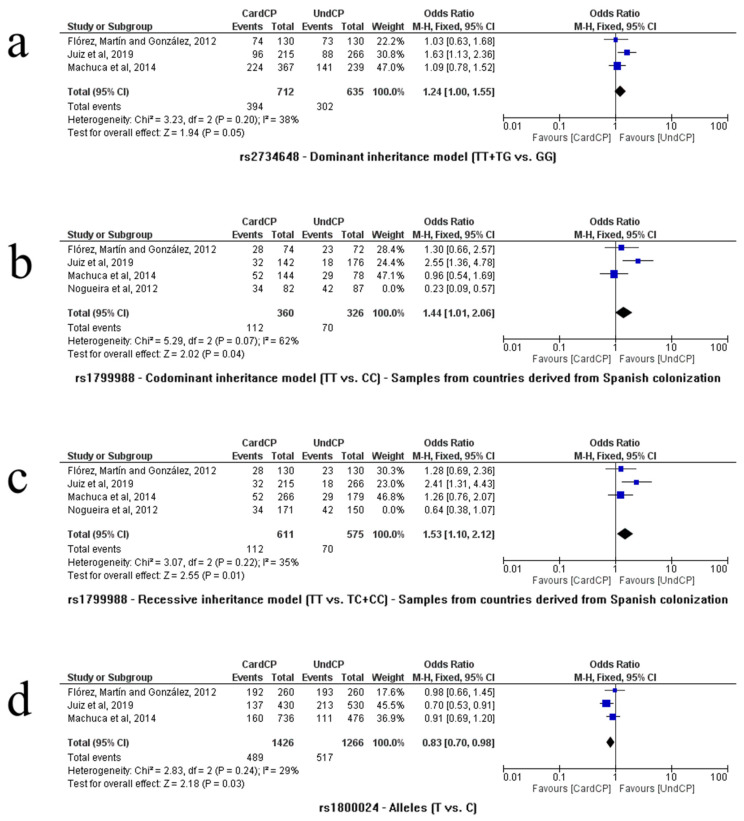
Meta-analysis of CCR5 genetic polymorphisms (rs2734648, rs1799988 and rs1800024) associated with CD. Comparisons were performed using dominance, codominance and recessiveness models. In our study, some analyses showed new associations based on investigated subgroups. The Mantel–Haenszel (M–H) test is the statistical method that was applied to generate an estimate of the association between Chagas disease and the analyzed risk factor after calculation adjustments [(**a**) dominant inheritance model TT+TG vs. GG, (**b**) codominant inheritance model TT vs. CC, (**c**) recessive inheritance model TT vs. TC+CC, and (**d**) alleles T vs. C]. References: Flórez, Martín and González, 2012 [10]; Juiz et al., 2019 [26]; Machuca et al., 2014 [28]; and Nogueira et al., 2012 [25].

The PCA showed in our analysis that PC1 corresponded to 70.91% of the variance, while PC2 corresponded to 21.16%, totaling 92.07% of the variance (Figure 4b). The variables that dominated in PC1 were the A and G alleles, and the A/A and G/G genotypes. On the other hand, the variable that dominated in PC2 was the A/G genotype. According to the PCA, the A allele and the A/A genotype were correlated, while the G allele and the G/G genotype were correlated. The A/G genotype was not correlated with other data in the multivariate analysis. Other correlations were not possible to establish by PCA, since there was a high degree of heterogeneity in some of the investigated groups, as pointed out by the meta-analysis.

#### 3.2.4. rs2734648

As shown in Figure 4a, three independent studies analyzed the relationship between the rs2734648 SNP and CD [10,24,28]. The comparison between cardiac patients vs. asymptomatic patients (n = 1347, CardCP = 712/UndCP = 635) was performed using these papers. Other comparisons were not possible. In the dominance model (T/T + T/G vs. G/G), an association was found between T carriers and the risk of developing the cardiac form (*p* = 0.05, OR = 1.24, 95% CI (1.00–1.55), I^2^ = 38%, fixed model). In the PCA, PC1 corresponded to 72.67% of the variance, while PC2 corresponded to 27%, totaling 99.67% of the variance (Figure 3c). The variables that dominated in PC1 were the T and G alleles, and the T/T and T/G genotypes. PC2 was dominated by the G allele and the T/T and G/G genotypes. It was observed that the T allele and the T/T and T/G genotypes were related, while the G allele and the G/G genotype were related, as shown in Figure 3.

#### 3.2.5. rs1799988

Flórez, Martín and González, Juiz et al., Machuca et al., and Nogueira et al. [10,25,26] evaluated the influence of the rs1799988 polymorphism on CD, and these studies were used to compare cardiac patients vs. asymptomatic (n = 1507, CardCP = 782/UndCP = 725). No differences were found between the groups in any genetic model. However, when the analysis delimited only patients whose countries originated from Spanish colonization (Flórez, Martín and González, 2012 [10]; Juiz et al. [26] and Machuca et al. [28], n = 1186, CardCP = 575/UndCP = 611), the codominance model (T/T vs. C/C) showed an association between the homozygous T/T genotype and the risk of developing the cardiac form of CD (*p* = 0.04, OR = 1.44, 95% CI (1.01–2.06), I^2^ = 62%, fixed model); similarly, the recessiveness model (T/T vs. T/C + C/C) also indicated an association of the homozygous T/T genotype with the same risk of developing the cardiac form of CD (*p* = 0.01, OR = 1.53, 95% CI (1.10–2.12), I^2^ = 35%, fixed model), as shown in Figure 4b and Figure 4c, respectively. Other statistical associations were not found.

#### 3.2.6. rs41469351

Only three studies involving patients from Spanish colonization countries analyzed the SNP rs41469351 [10,24,28]. Cardiac vs. asymptomatic patients were compared using data from these studies (n = 1347, CardCP = 712/UndCP = 635); however, due to the rarity of the T allele and therefore the T/T genotype (not found in any of the studies), further comparisons could not be conducted. For viable comparisons, no differences were found between the analyzed groups.

#### 3.2.7. rs1800024

Flórez, Martín and González, Juiz et al., and Machuca et al. [10,26,28] evaluated the influence of the SNP rs1800024 on CD. The comparison derived from these studies verified relationships between cardiac patients vs. asymptomatic (n = 1346, CardCP = 713/UndCP = 633). None of the evaluated genetic models showed any significant difference in the genotypic comparisons. However, the allele comparison (T vs. C) showed an association between the T allele and a decrease in the risk of developing the cardiac form of CD (*p* = 0.03, OR = 0.83, 95% CI (0.70–0.98), I^2^ = 29%, fixed model) (Figure 4d). The PCA was used to analyze the relationships between the data, where PC1 and PC2 presented, respectively, 77.88% and 21.57% of the variances, totaling 99.45%. PC1 was dominated by the C allele and the homozygous genotypes C/C and T/T, whereas PC2 was dominated by the T allele and the T/C genotype. Two-dimensional analysis indicated a correlation between the T allele and the T/T genotype, while the C allele and the T/C and C/C genotypes were correlated (Figure 3d).

#### 3.2.8. rs1800023

**Figure 5:**
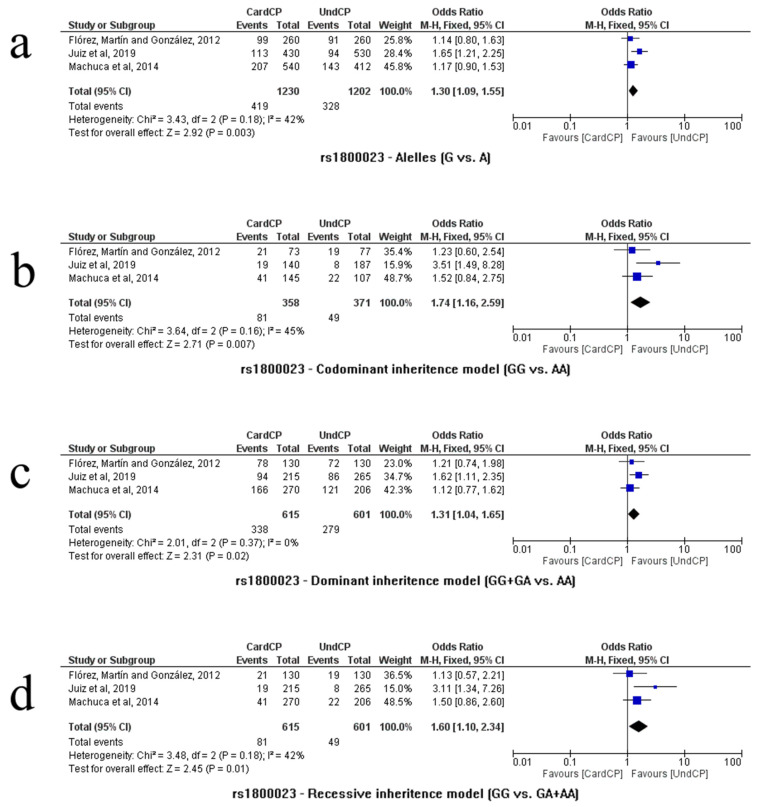
Meta-analysis of SNP rs1800023 (CCR5 genetic polymorphism) associated with CD. Analysis was performed on dominance, codominance and recessiveness models. The Mantel–Haenszel (M–H) test is the statistical method that was applied to generate an estimate of the association between Chagas disease and the analyzed risk factor after calculation adjustments. [(**a**) alleles G vs. A, (**b**) codominant inheritance model GG vs. AA, (**c**) dominant inheritance model GG + GA vs. AA, and (**d**) recessive inheritance model GG vs. GA + AA]. References: Flórez, Martín and González, 2012 [10]; Juiz et al., 2019 [26]; and Machuca et al., 2014 [28].

Only three studies analyzed the rs1800023 polymorphism [10,26,28] and genetic models comparing groups of cardiac vs. asymptomatic patients (n = 1216, CardCP = 615/UndCP = 601). The allele comparison (G vs. A) indicated an association of the G allele with the risk of developing the cardiac form of CD (*p* = 0.003, OR = 1.30, 95% CI (1.09–1.55), I^2^ = 42%, fixed model). Significant differences were observed in almost all genetic models. The risk of developing the cardiac form of CD was associated with the G/G homozygote in the codominance model (G/G vs. A/A): *p* = 0.01; OR = 1.53; 95% CI (1.10–2.12); I^2^= 35%; fixed model. The mutated G/G genotype was also associated in the recessiveness model (G/G vs. G/A + A/A): *p* = 0.01; OR = 1.60; 95% CI (1.10–2.34); I^2^ = 42%; fixed model. In the dominance model (G/G + GA vs. A/A), genotypes associated with the G allele were also associated with the risk of developing the cardiac form of CD (*p* = 0.02, OR = 1.31, 95% CI (1.04–1.65), I^2^ = 0%, fixed model). Representations of allelic and genotypic associations in the codominance, recessiveness and dominance models are shown in Figure 5a, Figure 5b, Figure 5c and Figure 5d, respectively.

PCA indicated variances of 56.00% and 42.48% for PC1 and PC2, respectively, totaling 98.48%. The G allele and the G/A and G/G genotypes dominated PC1, while the A allele and the A/A genotype dominated PC2. The analysis of the graphs indicates a correlation between the A allele and the A/A genotype, as well as a correlation between the G allele and the genotypes carried by this allele (G/G and G/A) (Figure 3e).

Following our analyses, no other association between the analyzed variants and CD was verified. Considering that all the analyses showed a sample power >80% (good sample representativeness), and with the exclusion of the SNPs rs333 and rs41469351 (unfeasible meta-analysis), it is possible to suggest that the other alleles and genotypes that did not show any association with CD are not factors capable of influencing this infection.

### 3.3. Publication Biases and Heterogeneity

As our analysis had less than ten studies in each comparison (K < 10), we considered inspecting the funnel-plot graphs to assess possible biases in the results of our comparisons. However, the scarcity of studies that analyzed the polymorphisms included in this review did not allow for a distribution that was considered sufficient to infer the existence of publication bias. In addition, studies were found on both sides of the funnel-plot graphs (Supplementary Figures S1 and S2), which may indicate symmetry and a lack of bias.

In our investigation, only a significant comparison of the rs2856758 SNP (CardCP vs. UndCP, G/A vs. A/A, codominance model) indicated substantial heterogeneity (I^2^ = 94%, *p* < 0.00001), as shown in Figure 2d. None of the other results showed significant heterogeneity.

### 3.4. SNP2TFBS

**Figure 6:**
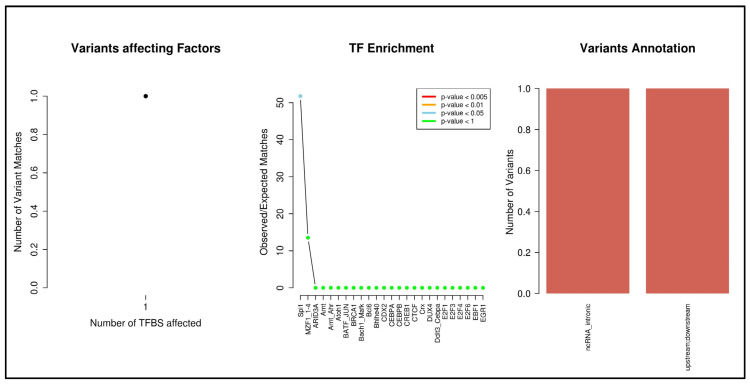
SNP2TFBS analysis of polymorphisms related to the CCR5 gene. Graphical representation requires at least two associations, and no other CCR5 polymorphisms have been listed as influencing any transcription factor. The rs1800629 SNP (TNF-α gene, upstream) was used for this purpose.

The prediction by the analysis tool indicated only one polymorphism (rs1800023, third intron) of the CCR5 gene capable of affecting the Spi1 transcription factor binding (*p* < 0.05), as represented in Figure 6.

### 3.5. Correlation between HHC Haplotype, Alleles and CD

After the quantitative analyses, we observed that all alleles directly or indirectly associated with the cardiac form of CD (rs2734648 (T), rs1799987 (G), rs1799988 (T) and rs1800023 (G) were the same ones that constituted a haplotype described in the literature as HHC. To verify the possible correlations between the alleles associated with the development of the cardiac form of CD and the haplotype, an exploratory analysis was performed between HHC and the associated alleles. For the analysis, information from the studies included in this review was used [10,26,28]. Quantitative data were extracted from the haplotypes observed in cardiac patients, as well as data from alleles that formed the haplotypes in cardiac and indeterminate patients. The extracted data were used to construct a Pearson correlation. As alleles are structurally related to the haplotype, there is a possibility of error in interpreting the results. In this way, we used a second haplotype known as HHE as a control.

Two conditions were considered in order to assume the correlation between HHC and its alleles (HHC–TGTG) with the development of the cardiac form of CD as true. First, all correlations between the presented alleles and HHC should be strong (>0.800) and positive, and second, the results should be maintained when adding data from the alleles of undetermined patients to the correlation. The inclusion of allelic data from undetermined patients in this second step aims to avoid misinterpretation of the correlation, given that the allele and HHC data came from the same groups of patients with the cardiac form. If both HHC–TGTG and HHE (HHE–GACA) are strongly positively correlated with their respective alleles, then it is suggested that the correlation between the alleles and haplotypes occurs due to structural dependence. However, if the pre-established correlations are observed only in HHC and its respective alleles, then a correlation with the development of the cardiac form of CD and HHC–TGTG is suggested. The correlation procedures were the same for both haplotypes.

All correlations between HHC and cardiac patient alleles were strongly and positively correlated (>0.950). These correlations were not found between HHE and its respective alleles. Considering the indeterminate patients in the analysis, all correlations between HHC and the alleles remained strong and positive: rs2734648-T (0.980, *p* > 0.001), rs1799987-G (0.935, *p* > 0.01), rs1799988-T (0.926, *p* > 0.01) and rs1800023-G (0.945, *p* > 0.01). For the tested conditions, the correlations between HHE and its respective alleles were classified as median or low (<0.700). Complementarily, using data from the articles, the total frequencies of HHC in cardiac and asymptomatic patients were compared using the χ2test. The results showed a significant difference between HHC frequencies between cardiac and asymptomatic patients (χ2 = 19.28, df = 1, *p* < 0.0001; OR = 1.8, 95% CI (1.38–2.34); SP = 100%; n = 1338, CardCp = 898/UndCP = 440), which suggests a correlation between HHC–TGTG and the cardiac form of CD.

## 4. Discussion

Chemokines are an important group of small cytokines (7–15 kDa) capable of specifically recruiting subgroups of leukocytes. There are two major subfamilies of chemokines based on the position of their cysteine residues: CXC (neutrophil chemotactics) and CC (monocyte chemotactics and lymphocyte subtypes) [7]. The CCR5 protein is a CC-type chemokine receptor that binds to molecules such as MIP1-α (CCL3), MIP1-β (CCL4) and RANTES (CCL5) [29,30], all related to inflammatory diseases, making CCR5 an important molecular target of these diseases, and often a common point between immunity and their pathophysiology [31,32,33].

CCR5 is expressed in monocytes, macrophages and lymphocytes such as TH1 cells (known to aid in the destruction of pathogens) [34]. In severe cases of CD in the cardiac form, the patient develops chronic Chagas cardiomyopathy (CCC), which is characterized by persistent myocarditis, with histologically detected tissue fibrosis, the destruction of cardiomyocytes, and a mononuclear infiltrate (usually T cells and macrophages) [25,35]. In this regard, such events suggest the involvement of CCR5 as a collaborative factor in chronic inflammation [35]. Chemokines and their receptors have been studied in CD due to their possible involvement in the chronic phase of the cardiac form, since they allow the maintenance of chronic inflammation, which in turn is the result of the inability of the immune system to completely eliminate the *T. cruzi* parasite [8,13].

Studies indicate that the chronic inflammation of the cardiac form of CD results in tissue damage and is caused by the attempt to eliminate the *T. cruzi* parasite. Cardiomyocytes produce cytokines, chemokines and nitric oxide that attract leukocytes to the inflammatory site, and thus promote the control of parasitic intracellular replication [9]. The expression of chemokine receptors in T cells associated with the expression of specific chemokines in tissues is an important factor that controls lymphocyte infiltration in different types of tissues [25,36]. Therefore, understanding the colonization mechanisms of the inflammatory response cells in cardiac tissues may reveal molecular targets that modulate the inflammatory response in the cardiac form of CD [8,13].

The CCR5 gene is one of the most studied genes in CD [4,37]. In vivo studies have shown that mice deficient in this gene do not develop the cardiac form, indicating that the expression of CCR5 is linked to the chronic cardiac form of the disease, and to the progression to CCC [8]. Interestingly, this evidence is supported by patient studies, in which analyses conducted with cardiomyopathic patients exhibited increased CCR5 expression, resulting in increased inflammation [13,25]. Studies on CCR5 are some of the most replicated, as well as studies on genetic variants of this gene, whose results are sometimes related to CD, especially with chronic forms of the disease [38].

In our work, we compiled the information present in the literature about the influence of genetic variants of the CCR5 gene on CD. In the qualitative synthesis, we observed that all studies were carried out with samples from South America, which points to the concern about the disease in that region. The patients’ parasitemia was scarcely evaluated in the works found. The lack of verification of parasitemia leaves a gap in the influence of the studied genetic variants. There was also a shortage of studies that analyzed the influence of CCR5 and its genetic variants on the digestive form of the disease, since only one study analyzed this relationship [13].

For the construction of the groups used in the comparisons in the quantitative synthesis, we observed that the authors of the studies used different criteria to group the participants as chronic cardiac patients (Table 1). We observed that the main criteria used were the verification of mechanical and morphoanatomical abnormalities of the cardiac structures, and the electrocardiogram consistent with Chagas cardiomyopathy. We could assume that there was homogeneity in this group, so we presented the criteria used in the studies and generically classified them as the “CardCP” group.

The most notorious polymorphism studied in the works was rs1799987 (59029A/G). In the quantitative synthesis, by comparing the CardCp and nonCP groups, our analyses showed that the A/G genotype was associated with the risk of progression to the cardiac form. However, it is important to highlight that the comparison between infected patients and healthy controls presents a non-optimized experimental design, since the comparison is based on a control group that has no chance of evolving to the cardiac form, which would be optimized by the comparison with asymptomatic infected patients (UndCP), allowing a comparison between groups that are in the same infectious condition but presenting forms that evolve in different ways [11].

Our global comparison between CardCp and UndCP did not indicate an association with rs1799987; however, the subgroup of patients from countries originating from Spanish colonization indicated an association of the G/G genotype (recessive inheritance model) with the risk of developing the cardiac form, contradicting the study by Calzada et al. [9], which indicated that the G allele was more present in asymptomatic individuals. It is possible that the genetic background of the population used by Calzada and collaborators [9] was not similar to the populations of the other studies, and therefore they did not find these results. Another possibility is that the sample power of the study developed by Calzada and collaborators [9] was not ideal for comparison, resulting in a false positive. Our sample power in all positively associated results was >80%, indicating representativeness of the population (Table 1).

The rs2856758 SNP A/G genotype in the codominance model was associated with a decreased risk of developing the cardiac form of CD. This result was not observed in other studies [10,26,28];, however, Flórez et al. [10] observed an increased G allele in the asymptomatic group compared to the cardiomyopathic, associating this allele with a decrease in the risk of developing the cardiac form of CD. Our quantitative synthesis also showed an association between the rs2734648 T/T and T/G genotypes and the risk of developing the cardiac form of patients. Flórez et al. [10] have already observed an association of the T allele of this polymorphism with a reduction in the risk for the severity of Chagas cardiomyopathy, also corroborating our results.

Our global analysis of the rs1799988 polymorphism did not find differences between the analyzed groups, but in the analysis of the subgroup of countries formed after Spanish colonization [10,26,28], the codominance model showed an association between the homozygous T/T genotype and the cardiac form of CD, while the recessiveness model associated the same T/T genotype with the cardiac form. Unlike our findings, Nogueira et al. [25] identified an increased C/C genotype and C allele in the cardiac group (CCC) compared to the moderate group. However, Nogueira et al. [25] used a Brazilian sample, while our subgroup results did not include Brazilians. It is important to emphasize that the countries of South America have populations with a high genetic mix, coming from native Amerindian, African and European populations. The Brazilian population, on the other hand, has a high contribution of European and African genomic inheritance, and a smaller Amerindian contribution. Thus, the samples from countries used in the quantitative synthesis of our study have greater contributions from European and Amerindian genomic heritages [39], which may result in different epidemiological scenarios.

The SNP rs1800023 indicated four different associations, all to some degree related to the G allele when comparing CardCP vs. UndCP (Figure 4). Machuca and others [28] have already established a relationship between the G allele and some degree of CCC severity, supporting our results. The rs1800024 SNP indicated the T allele as a risk factor for cardiomyopathy in CD, also previously suggested by Juiz and coauthors [26], although different from the results of Machuca et al. [28], which related the allele with the severity of the disease.

In this work, we used principal component analysis (PCA), an exploratory statistical analysis that establishes relationships between data without the prior definition of a model, unlike the meta-analysis, which was based on standardized models of genetic inheritance. These relationships are important in order to evaluate how the interaction of alleles and genotypes occurs in the samples. According to PCA, it is possible to observe data dispersion, which may not be fully represented in analyses using pre-established models, as in the meta-analysis performed in our study. Unfortunately, few inferences could be obtained using PCA due to the small number of available studies and, consequently, the limited data. In the PCA, the graphs (Figure 3) and/or three-dimensional representations eventually presented correlations using alleles and genotypes that grouped and curiously resembled the phenotypic groups, as well as referring to some patterns of genetic inheritance. However, these groupings did not necessarily reflect the possible phenotypic groups.

The PCA of rs1799987, based on the interaction between the genotypes, alleles and the condition of the participants (CardCp vs. nonCP), indicated that there was a correlation between the G carriers (G/A and G/G) and the G allele, as well as a correlation between the A allele and the AA genotype. The analysis performed using the SNP rs2856758 reported a cluster formed between the A allele and the A/A genotype, and a second cluster formed by the G allele and the G/G genotype, while the A/G genotype did not seem to be related to any of these groups. Additionally, it should be noted that in our study the A/G genotype obtained an association in the codominant inheritance model. Interestingly, this graphic representation was similar to the phenotypes that were presented in genetic inheritance in the codominance model, that is, the presentation of three different profiles.

The T/T and T/G genotypes of the rs2734648 SNP were both associated with the development of the cardiac form in the dominant inheritance model. In the multivariate analysis, these two genotypes were correlated with the T allele, while the G allele and the G/G genotype formed a second cluster in the PCA. This graphical representation is also similar to the phenotypic profiles in dominant/recessive-type inheritance (group with dominant allele trait and group with recessive allele trait). Our observations from the multivariate analysis of the rs1800023 SNP indicated a correlation between the A allele and the A/A genotype. Despite being relatively distant, a correlation was also observed between the G allele and the G/G and G/A genotypes (Figure 3). Finally, the PCA of the SNP rs1800024 indicated two clusters, one between the T allele and the T/T genotype, and another between the C allele and the T/C and C/C genotypes, similar to the phenotypic profiles observed in the inheritance of the dominant/recessive type.

The SNP2TFBS tool was used to verify the influence of polymorphisms in this review on transcription factors. The rs1800023 SNP was shown to be able to influence Spi1 factor activity. Spi1 is an important transcription factor that binds to a sequence known as PU-box (a purine-rich DNA sequence), located near the promoter region of target genes. Thus, Spi1 co-regulates the expression of target genes together with other transcription factors and cofactors. The Spi1 protein can also bind to RNA to regulate the selective splicing of target genes [40].

Mummidi et al. [40] have already predicted through algorithms the action of Spi1 in the promoter region of CCR5; however, it is not exactly influenced by SNP rs1800023. Although they were unable to determine the specific binding of a transcription factor to rs1800023, they were able to determine that there was a factor that bound to the G allele but not the A allele, most likely Spi1. According to Mummidi and collaborators [40], the binding of the transcription factor to the altered regulatory regions can be an important selection pressure factor caused by the inter-relationship of the host with the parasite, which can determine the final result of the infection. In addition to their work evaluating the role of transcription factors in the promoter, the study by Mummidi et al. also determined the presence of nine human haplotypes (HHs) and evaluated their functionality in gene expression through their respective ability to bind to different transcription factors.

Many of the polymorphisms analyzed in this review are constituents of CCR5 haplotypes and capable of influencing its expression. Interestingly, all our findings involving an association with the risk of developing Chagas cardiomyopathy are within the C haplotype (HHC), according to Mummidi et al.: rs2734648 (T), rs1799987 (G), rs1799988 (T) and rs1800023 (G), this haplotype seems to induce a low transcriptional activity in Jukart cells (T lymphocyte lineage derived from leukemia), while in Nk cells (CD56+) and CD16+ it presents a high activity [41,42]. The studies by Flórez et al., Machuca et al. and Juiz et al. [10,26,28]—the latter with participants defined as creole and non-endemic—observed that HHC was the most common haplotype in their samples.

To verify possible correlations between HHC and its alleles (HHC–TGTG) and the cardiac form of CD, a Pearson correlation was performed. Our findings demonstrated that there was a very strong correlation between HHC and alleles. All the correlations were positive and significant (>0.800; *p* < 0.05). These results were not found in the correlation study using HHE as a control. Thus, we assume the relationship between HHC–TGTG and the development of the cardiac form of CD to be true. This result is supported by the fact that there was a significant difference in the frequency of HHC between the cardiac and indeterminate groups.

There are different hypotheses to explain the development of the cardiac form of CD in a certain group of patients, such as the influence of the genetic variability of the infecting parasite, molecular mimicry (between parasite antigens and host proteins, resulting in an autoimmune reaction by cross-reaction and reactivating cells), localized damage (induced by in situ expression of a tissue rich in infiltrate, producing chemokine receptor ligands), and finally, the patient’s genetics [6,25,43].

Among the hypotheses described, we believe that the patient’s genetics is the one that best explains the development of the cardiac form, since studies have indicated the lack of influence between the genetic variability of the parasite and the development of this form of CD [12,44]. Furthermore, any in situ reaction depends on molecular pathways that can be influenced by the patient’s genotype, which makes this last hypothesis a result of the patient’s genetic influence.

According to the authors of previous studies, it is possible that the course of CD in a patient is not the result of the mere presence or absence of a single genetic variant. It is believed that combinations between several different variants in several genes modestly contribute to the pathogenesis of the disease [10,26,28]. The CCR5 gene has a strong linkage disequilibrium in its shared loci with the CCR2 gene, which allowed us to verify the influence of multiple polymorphic changes in CCR5 expression on the cell surface as the CCR5 expression rate in blood cells changed [41,42]. Our work statistically contributes to this prerogative, indicating not only the individual influence of certain polymorphisms, but also the haplotypic configuration, which influences the migration and accumulation of cells, increasing the cellular infiltrate and leading to the development of the cardiac form of CD.

## 5. Conclusions

Some limitations of this study can be highlighted. Our quantitative synthesis presents results based on the included studies. These studies used different methodologies, as well as different comparison groups, which may imply inconsistencies in our results. It was also not possible to analyze the influence of environmental factors, such as the exposure time of patients, or factors intrinsic to the participants, such as age and gender. The scarcity of data was a limitation for our analyses and, consequently, our inferences. However, despite these limitations, carrying out this study had advantages, such as a better understanding of CD.

In short, we emphasize that meta-analysis is a tool capable of narrowing the relationship between the genetic profile of the hosts and the clinical outcome. In our analysis, the rarity of the Δ32 alleles of the SNP rs333 and the T allele of the SNP rs41469351 was evident in the studied samples from South American countries. In addition, the C allele (rs1800024) and the A/G heterozygote (rs2856758) were associated with a reduced risk of developing the cardiac form of CD. All alleles associated with an increased risk of developing the cardiac form of CD comprised human haplotype C (HHC). Our HHC–TGTG analysis was also shown to be correlated with the cardiac form of CD. In general terms, exploratory statistical analysis, such as PCA, made it possible to infer correlations between different factors without the need to establish genetic models. Based on our findings, PCA appears to be a strong tool that can help in the understanding of infectious diseases such as CD. Due to the heterogeneity of the data, we advise that the inferences indicated here be analyzed with caution.

## Figures and Tables

**Table 1:** Data extracted from articles included in the systematic review. The data presented consist of: STREGA score, main results associated with CCR5 variants and overall frequency (calculated based on the sum of samples from all collected studies), inclusion in the quantitative synthesis (meta-analysis), types of groups compared in the quantitative synthesis, and number of participants in each group (NonCP, CP, SympCP, CardCP and UndCP).

Study Characterization									
References	Study Country ^1^	Study Design	Population/Sample (n)	Group Characterization ^2^	STREGA Score	Confirmation Method of *T. cruzi* Infection	Confirmation Method of Polymorphism	Parasitemia Evaluation	Hardy-Weinberg Equilibrium (HWE) ^3^
Oliveira et al., 2014 [24]	Brazil	Cross-sectional	Brazilians/n = 168	CP = 168 (CardCP = 168)	8—Low quality	ELISA	PCR-RFLP	No	Nd
Frade et al., 2013 [11]	Brazil	Cohort	Brazilians/n = 433	CP = 433 (CardCP = 315 and UndCP = 118)	13—Medium quality	ELISA, IHA and IIF	Hybridization assay and qPCR	No	Yes ^4^
Nogueira et al., 2012 [25]	Brazil	Cohort	Brazilians/n = 321	CP = 22 (CardCP = 171) and UndCP = 150	12—Medium quality	ELISA, IHA and IIF	qPCR	No	Yes
Juiz et al., 2019 [26]	Argentina	Case-control	Argentinians/n = 480	CP = 480 (CardCP= 215 and UndCP = 256)	14—Medium quality	Serologically	qPCR	No	Yes ^5^
Calzada et al., 2001 [9]	Spain	Case-control	Peruvians/n = 172	CP = 172 (UndCP = 53; CardCP = 32) and nonCP = 87	14—Medium quality	ELISA, IHA and IIF	PCR-RFLP	No	Yes
Lima et al., 2018 [12]	Brazil	Observational study—case series	Brazilians/n = 6	CP = 6 (UndCP = 1 and CardCP = 5)	10—Medium quality	ELISA, IHA and PCR	PCR and PCR-RFLP	No	Nd
Batista et al., 2018 [8]	Brazil	Cross-sectional	Brazilians/n = 406	CP = 406 (CardCP = 296 and UndCP = 110)	17—High quality	ELISA and IIF	qPCR	Yes	Nd
De Oliveira et al., 2015 [13]	Brazil	Case-control	Brazilians/n = 412	CP = 240 (CardCP = 121 and DigCP = 98) and nonCP = 172	15—Medium quality	ELISA	PCR and PCR-RFLP	No	Yes
Flórez; Martín; González, 2012 [10]	Colombia	Case-control	Colombians/n = 260	CP = 260 (UndCp = 130 and CardCP = 130)	16—Medium quality	ELISA and IHA	PCR and PCR-RFLP	No	Yes
Fernandez-Mestre; Montagnani; Layrisse, 2004 [27]	Venezuela	Cross-sectional	Venezuelans n = 107	CP = 107 (UndCP = 34 and CardCP = 73)	11—Medium quality	ELISA, IHA and IIF	PCR-RFLP	No	Nd
Machuca et al., 2014 [28]	Colombia	Cohort	Colombians/n = 476	CP = 476 (UndCP = 206 and CardCP = 270)	13—Medium quality	ELISA and IHA	qPCR	No	Yes
**CCR5 Polymorphisms**									
**Polymorphism**	**Mutated Nucleotide Localization/Variation Type [and Change]/Consequence ^6^**		**Main Finds of Studies**						**Quantitative Synthesis by Meta-Analysis (Yes/No)**
rs2856758	46370170/SNV [A > G]/intron variant, 5 prime UTR variant		Flórez et al., 2012 [10]: G allele increased in asymptomatic group in a comparison with the cardiomyopathy group, indicating the cardiomyopathy risk reduction (*p* = 0.021, OR = 0.51, 95% CI (0.29–0.91)). Juiz et al., 2019 [26]: not associated. Machuca et al., 2014 [28]: not associated.						Yes
rs2734648	46370349/SNV [G > C/G > T]/intron variant		Flórez et al., 2012 [10]: T allele associated with reduced risk of cardiomyopathy progression compared to symptomatic subgroups (group II vs. group III, *p* = 0.018, OR = 0.44, 95% CI (0.22–0.88)/and group III vs. group IV, *p* = 0.004, OR = 0.29, 95% CI (0.12–0.68)). Juiz et al., 2019 [26]: not associated. Machuca et al., 2014 [28]: not associated.						Yes
rs1799987	46370444/SNV [A > G]/intron variant		Batista et al., 2018 [8]: not associated. Calzada et al., 2001 [9]: G/A increased in asymptomatic group in a comparison with the cardiomyopathy group (*p* = 0.02, OR = 0.33, 95% CI (0.10–0.94)); G allele increased in asymptomatic group in a comparison with the cardiomyopathy group (*p* = 0.02, OR = 0.35, 95% CI (0.12–0.96)). Fernández-Mestre et al., 2004 [27]: not associated. Flórez et al., 2012 [10]: not associated. Juiz et al., 2019 [26]: not associated. Lima et al., 2018 [12]: not associated. Oliveira et al., 2015 [13]: A/A frequency was different among patients with digestive and cardiac forms, and health controls (*p* = 0.036, χ2 = 6.656 (DF = 2)); A/A frequency was different between patients with digestive and cardiac forms (*p* = 0.013, χ2 = 6.129 (DF = 1)); A/A frequency was different between patients with cardiac form and health controls (*p* = 0.077, χ2 = 3.128 (DF = 1)); Oliveira et al., 2014 [24]: not associated. Machuca et al., 2014 [28]: not associated.						Yes
rs1799988	46370768/SNV [C > T]/intron variant, 5 prime UTR variant		Flórez et al., 2012 [10]: not associated. Juiz et al., 2019 [26]: not associated. Machuca et al., 2014 [28]: not associated. Nogueira et al., 2012 [25]: C/C frequency was increased in CCC severe group than CCC moderate group (*p* = 0.01, χ2 = 5.55, OR = 2.31, 95% CI (1.14–4.67)); C allele was higher in CCC severe group than CCC moderate group (*p* = 0.01, χ2 = 6.15, OR = 0.58, 95% CI (0.37–0.89)).						Yes
rs41469351	46370771/SNV [C > T]/intron variant, 5 prime UTR variant		Flórez et al., 2012 [10]: not associated. Juiz et al., 2019 [26]: T allele frequency in a sample with Caucasian genetic background was increased in a demonstrated cardiomyopathy group than non-demonstrated cardiomyopathy group (*p* = 0.028, OR = 4.88, 95% CI (1.03–23.24)). Machuca et al., 2014 [28]: not associated.						Yes
rs1800023	46370817/SNV [A > G/A > T]/intron variant, 5 prime UTR variant		Flórez et al., 2012 [10]: not associated. Juiz et al., 2019 [26]: not associated. Machuca et al., 2014 [28]: G allele was more frequent in the group with lower CCC severity compared to symptomatic subgroups (group II vs. group III, *p* = 0.05, OR = 0.70, 95% CI (0.49–1.00)).						Yes
rs1800024	46371068/SNV [C > T]/intron variant		Flórez et al., 2012 [10]: not associated. Juiz et al., 2019 [26]: T allele associated with reduced risk of cardiomyopathy in the group without symptoms of cardiomyopathy (asymptomatic group) when compared to the group with symptoms of cardiomyopathy (cardiomyopathy group) (*p* = 0.041, OR = 0.69, 95% CI (0.49–0.99)). Machuca et al., 2014 [28]: T allele associated with higher disease severity when compared to symptomatic subgroups (group II vs. group III, *p* = 0.02, OR = 1.70, 95% CI (1.06–2.71)).						Yes
rs3176763	46372790/SNV [G > A/G > T]/intron variant		Frade et al., 2013 [11]: C/C frequency was increased in CCC group than asymptomatic group when considered gender (*p* = 0.006, OR = 1.79, 95% CI (1.18–2.70)); C/A frequency was increased in CCC group than asymptomatic group when considered left ventricular ejection fraction under 0.4% values (*p* = 0.005, OR = 1.88, 95% CI (1.20–2.94)).						No
rs333[Δ32]	46373453–46373487/frameshift variant (Delins, length 35 pb), coding sequence variant, intron variant		Calzada et al., 2001 [9]: not associated. Fernández-Mestre et al., 2004 [27]: not associated. Flórez et al., 2012 [10]: not associated. Oliveira et al., 2015 [13]: not associated.						Yes
rs3087253	46377198/SNV [C > T]/intron variant		Frade et al., 2013 [11]: not associated.						No
rs11575815	46378679/SNV [A > T]/intron variant		Frade et al., 2013 [11]: A/T and A/A frequecy was increased in the CCC group than asymptomatic group (*p* = 0.030, OR = 1.41, 95% CI (1.03–1.92)).						No

ELISA—enzyme-linked immunosorbent assay; IHA—indirect hemagglutination assay; IIF—indirect immunofluorescence; ^1^ country of study is based on the first author; ^2^ the characterization of each study was performed by us in order to make comparisons in the quantitative synthesis. The categorizations originally made by the authors of each article are described in Table 2; ^3^ Nd = HWE not described by the authors; ^4^ HWE was presented only for the control group; ^5^ rs1799864 and 1800024 are not in HWE; ^6^ information provided by NCBI.

**Table 2:** Description of the groups that constitute the CardCP grouping.

Reference	Description of CardCP Group Patients
Batista et al., 2018 [8]	Group in stage B1 (with structural heart disease, evidenced by ECG or ECHO, but with normal global ventricular function and neither current nor previous signs or symptoms of congestive heart failure) and group in stage C (with ventricular dysfunction and current or previous symptoms of congestive heart failure)
Calzada et al., 2001 [9]	Cardiomyopathic group with cardiac symptoms, which were assessed by clinical and electrocardiographic (ECG) characteristics compatible with chagasic cardiomyopathy
Fernandez-Mestre, Montagnani and Layrisse, 2004 [27]	Group B (arrhythmia-related symptoms or embolic episodes as a first symptom, and in whom radiological size ranged from normal to severe cardiomegaly and various arrhythmias) and group C (with overt congestive heart failure, most with severe cardiomegaly and various arrhythmias)
Flórez, Martín and González, 2012 [10]	Group II (radiology indicative of light heart hypertrophy or minor ECG alterations), group III (moderate heart hypertrophy and considerable ECG alterations, mainly advanced conduction abnormalities) and group IV (severe cardiomegaly and marked ECG alterations, predominantly frequent and/or complex forms of ventricular arrhythmia)
Juiz et al., 2019 [26]	CD group (with clinical symptoms and electrocardiography alterations)
Lima et al., 2018 [12]	Patients that presented cardiac arrhythmia with stimulus conduction disorder by EKG, compatible with chagasic cardiopathy
Machuca et al., 2014 [28]	Symptomatic group (with minor symptoms and alterations) and cardiomyopathic group (with considerable symptoms and alterations)
Nogueira et al., 2012 [25]	CCC (moderate group and severe group)
Oliveira et al., 2015 [13]	Group of cardiac form (CCHD when presenting with electrocardiographic or echocardiographic abnormalities consistent with the disease)

## Data Availability

Not applicable.

## Supplementary Materials

The following supporting information can be downloaded at: https://www.mdpi.com/article/10.3390/life13081677/s1, Figure S1. Funnel plots obtained from comparisons with significant results (*p* ≤ 0.05) of the SNPs rs1799987, rs2856758, rs2734648, rs1799988 and rs1800024. Figure S2. Funnel plots obtained from comparisons with significant results (*p* ≤ 0.05) of SNP rs1800023. Table S1. Genetic variants, comparisons made between groups and their individual and total frequencies.
