# Supplementary material for: Narrowing the Relationship between Human CCR5 Gene Polymorphisms and Chagas Disease: Systematic Review and Meta-Analysis
Source: Life (Basel). 2023 Aug 2;13(8):1677. doi: 10.3390/life13081677 (PMC10455882; doi:10.3390/life13081677)
Supplement: Supplementary file 1 [file life-13-01677-s001.zip › life-2440393-supplementary.pdf]

Supplementary Materials

Supplementary Figure S1. Funnel plots obtained from comparisons with significant results (p≤0.05) of the SNPs rs1799987, rs2856758, rs2734648, rs1799988 and rs1800024.

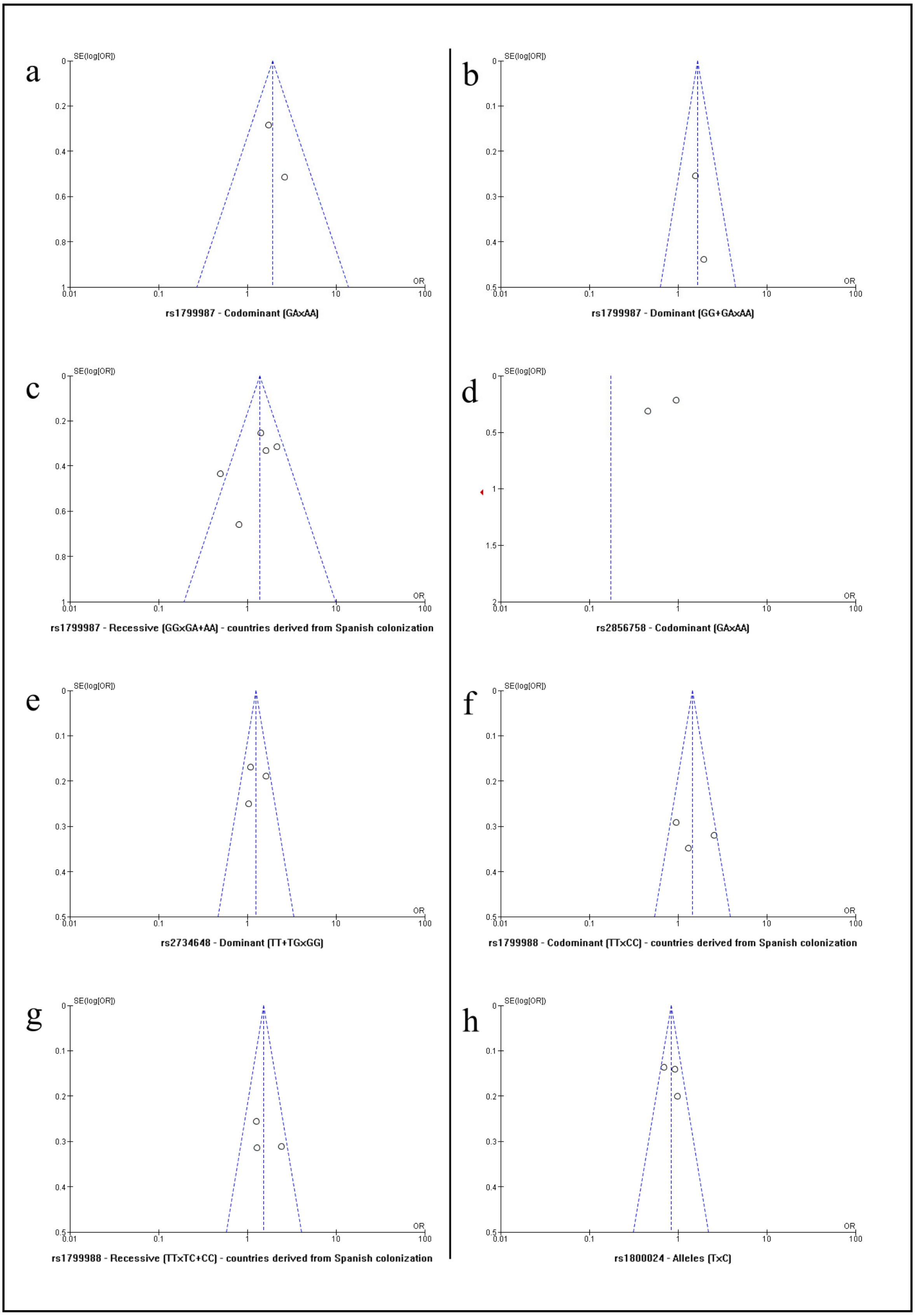

Source: Author, 2023.

Supplementary Figure S2. Funnel plots obtained from comparisons with significant results ($p \leq 0.05$) of SNP rs1800023.

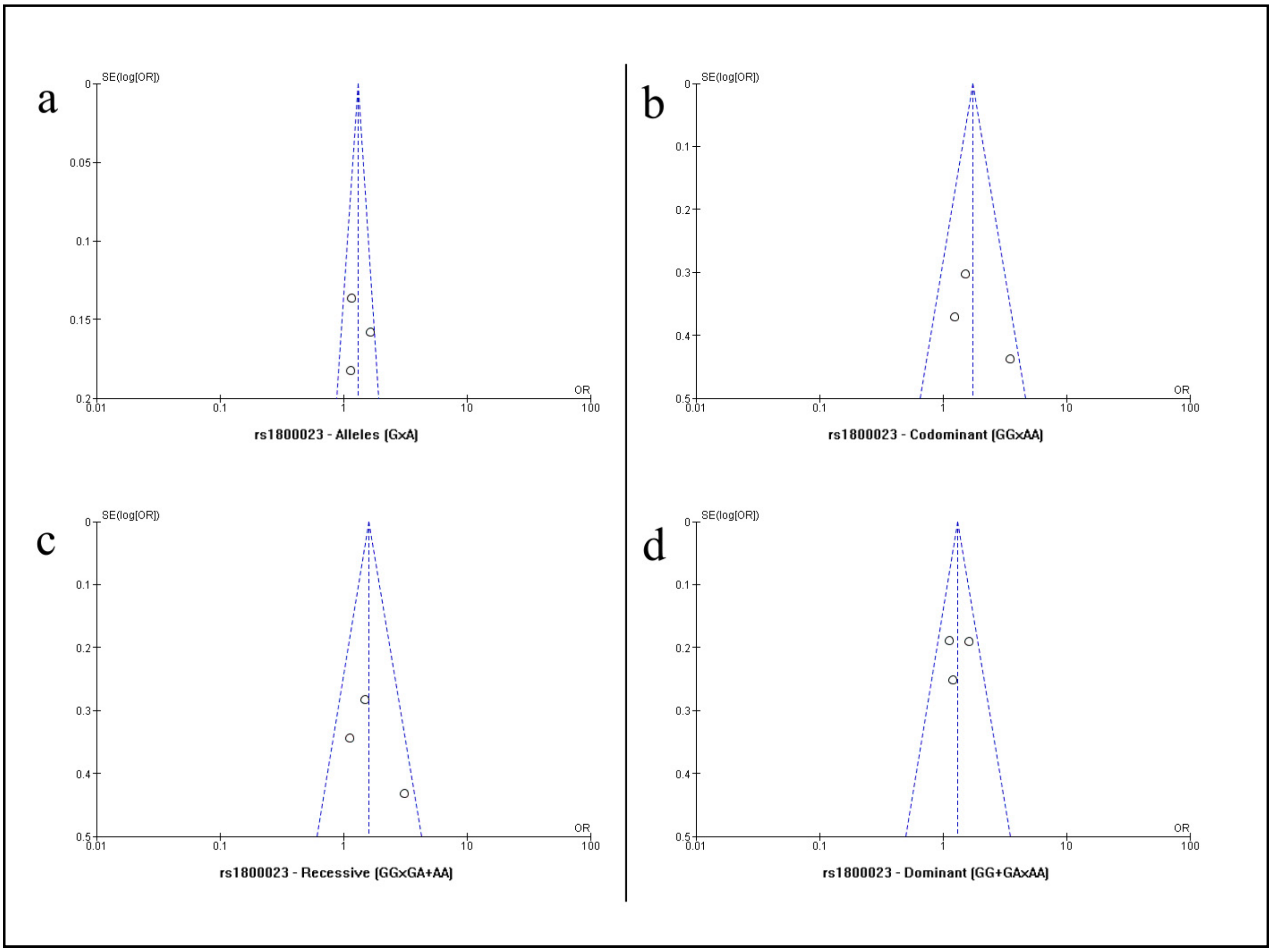

Source: Author, 2023.

In all groups, the Hardy-Weinberg equilibrium (HWE) was analyzed. The Chi-square test (χ^2) was performed in all comparisons, and results with $p \leq 0.05$ were considered significant.

Supplementary Table S1. Genetic variants, comparisons made between groups and their individual and total frequencies.

Polymorphism	Comparison (group 1 *vs*. group 2), and sample power (SP%)*	Group 1 frequency**	Group 2 frequency**	Total frequency (group 1+ group 2)**
rs2856758	CardCP *vs*. UndCP, SP=100%	AA=0,7599388379	AA=0,4076539101	AA=0,5912350598
		AG=0,1605504587	AG=0,2013311148	AG=0,1800796813
		GG=0,07951070336	GG=0,391014975	GG=0,228685259
		A=0,8402140673	A=0,5083194676	A=0,6812749004
		G=0,1597859327	G=0,4916805324	G=0,3187250996
rs2734648	CardCP *vs*. UndCP, SP>99%	TT=0,1348314607	TT=0,09921259843	TT=0,1180400891
		TG=0,4185393258	TG=0,3763779528	TG=0,3986636971
		GG=0,4466292135	GG=0,5244094488	GG=0,4832962138
		T=0,3441011236	T=0,2874015748	T=0,3173719376
		G=0,6558988764	G=0,7125984252	G=0,6826280624
rs1799987	nonCp *vs*. CardCP, SP>96%	AA=0,3661417323	AA=0,4444444444	AA=0,4307856761
		AG=0,4212598425	AG=0,3137254902	AG=0,4783538215
		GG=0,2125984252	GG=0,2418300654	GG=0,2266167825
		A=0,5767716535	A=0,6013071895	A=0,6699625869
		G=0,4232283465	G=0,3986928105	G=0,4657936932
	CardCP *vs*. UndCP, SP>99%	AA=0,373284538	AA=0,4380952381	AA=0,4014485256
		AG=0,4226898445	AG=0,4107142857	AG=0,4174857734
		GG=0,2040256176	GG=0,1511904762	GG=0,181065701
		A=0,5846294602	A=0,643452381	A=0,6101914123
		G=0,4153705398	G=0,356547619	G=0,3898085877
	CardCP *vs*. UndCP, SP>99%**	AA**=0,4106145251	AA**=0,4622093023	AA**=0,4358974359
		AG**=0,406424581	AG**=0,4098837209	AG**=0,4081196581
		GG**=0,1829608939	GG**=0,1279069767	GG**=0,155982906
		A**=0,6138268156	A**=0,6671511628	A**=0,639957265
		G**=0,3861731844	G**=0,3328488372	G**=0,360042735

	CardCP *vs.* UndCP, SP>37%***	AA***=0,3023872679	AA***=0,3289473684	AA***=0,3100189036
		AG***=0,4535809019	AG***=0,4144736842	AG***=0,4423440454
		GG***=0,2440318302	GG***=0,2565789474	GG***=0,247637051
		A***=0,5291777188	A***=0,5361842105	A***=0,5311909263
		G***=0,4708222812	G***=0,4638157895	G***=0,4688090737
	CP *vs.* nonCp, SP >37%	AA=0,3717105263	AA=0,3661417323	AA=0,3691756272
		AG=0,3914473684	AG=0,4212598425	AG=0,4050179211
		GG=0,2368421053	GG=0,2125984252	GG=0,2258064516
		A=0,5674342105	A=0,5767716535	A=0,5716845878
		G=0,4325657895	G=0,4232283465	G=0,4283154122
	SympCP *vs.* UndCP, SP=100%	AA=0,3346265761	AA=0,4380952381	AA=0,3810796366
		AG=0,4296799224	AG=0,4107142857	AG=0,4211651523
		GG=0,2356935015	GG=0,1511904762	GG=0,1977552111
		A=0,5494665373	A=0,643452381	A=0,5916622127
		G0,4505334627	G0,356547619	G=0,4083377873
	nonCP *vs.* UndCP, SP>57%	AA=0,3661417323	AA=0,4224137931	AA=0,3837837838
		AG=0,4212598425	AG=0,3534482759	AG=0,4
		GG=0,2125984252	GG=0,224137931	GG=0,2162162162
		A=0,5767716535	A=0,599137931	A=0,5837837838
		G=0,4232283465	G=0,400862069	G=0,4162162162
rs1799988	CardCP *vs.* UndCP, SP>99%	TT=0,1867007673	TT=0,1544827586	TT=0,1712010617
		TC=0,4347826087	TC=0,4303448276	TC=0,4326476443
		CC=0,378516624	CC=0,4151724138	CC=0,396151294
		T=0,4040920716	T=0,3696551724	T=0,3875248839
		C=0,5959079284	C=0,6303448276	C=0,6124751161
	CardCP *vs.* UndCP, SP>99%**	TT**=0,1833060556	TT**=0,1217391304	TT**=0,1534569983
		TC**=0,410801964	TC**=0,4330434783	TC**=0,4215851602
		CC**=0,4058919804	CC**=0,4452173913	CC**=0,4249578415
		T**=0,3887070376	T**=0,3382608696	T**=0,3642495784

		C**=0,6112929624	C**=0,6617391304	C**=0,6357504216
rs41469351	CardCP *vs*. UndCP#	TT=0<br>TC=0,01966292135<br>CC=0,9803370787<br>T=0,009831460674<br>C=0,9901685393	TT=0<br>TC=0,01417322835<br>CC=0,9858267717<br>T=0,007086614173<br>C=0,9929133858	TT=0<br>TC=0,01707498144<br>CC=0,9829250186<br>T=0,00853749072<br>C=0,9914625093
rs1800023	CardCP *vs*. UndCP, SP=100%	AA=0,4504065041<br>AG=0,4178861789<br>GG=0,1317073171<br>A=0,6593495935<br>G=0,3406504065	AA=0,5357737105<br>AG=0,3826955075<br>GG=0,08153078203<br>A=0,7271214642<br>G=0,2728785358	AA=0,4925986842<br>AG=0,4004934211<br>GG=0,1069078947<br>A=0,6928453947<br>G=0,3071546053
rs1800024	CardCP *vs*. UndCP, SP>99%	TT=0,1795231417<br>TC=0,3267882188<br>CC=0,4936886396<br>T=0,3429172511<br>C=0,6570827489	TT=0,2353870458<br>TC=0,345971564<br>CC=0,4186413902<br>T=0,4083728278<br>C=0,5916271722	TT=0,205794948<br>TC=0,3358098068<br>CC=0,4583952452<br>T=0,3736998514<br>C=0,6263001486
rs333[Δ32]	CP *vs*. nonCp#	Del/Del=0<br>Del/Ins=0,06647398844<br>Ins/Ins=0,9335260116<br>Del=0,03323699422<br>Ins=0,9667630058	Del/Del=0,003861003861<br>Del/Ins=0,06177606178<br>Ins/Ins=0,9343629344<br>Del=0,03474903475<br>Ins=0,9652509653	Del/Del=0,001051524711<br>Del/Ins=0,06519453207<br>Ins/Ins=0,9337539432<br>Del=0,03364879075<br>Ins=0,9663512093
	CardCP *vs*. UndCP#	Del/Del=0<br>Del/Ins=0,07103825137<br>Ins/Ins=0,9289617486<br>Del=0,03551912568<br>Ins0,9644808743	Del/Del=0<br>Del/Ins=0,06912442396<br>Ins/Ins=0,930875576<br>Del=0,03456221198<br>Ins=0,965437788	Del/Del=0<br>Del/Ins=0,07032590051<br>Ins/Ins=0,9296740995<br>Del=0,03516295026<br>Ins=0,9648370497
	nonCP *vs*. UndCP#	Del/Del=0,003861003861<br>Del/Ins=0,06177606178<br>Ins/Ins=0,9343629344<br>Del=0,03474903475	Del/Del=0<br>Del/Ins=0,06912442396<br>Ins/Ins=0,930875576<br>Del=0,03456221198	Del/Del=0,002100840336<br>Del/Ins=0,06512605042<br>Ins/Ins=0,9327731092<br>Del=0,03466386555

	Ins=0,9652509653	Ins=0,965437788	Ins=0,9653361345
nonCp *vs.* CardCP#	Del/Del=0,003861003861	Del/Del=0	Del/Del=0,0016
	Del/Ins=0,06177606178	Del/Ins=0,07103825137	Del/Ins=0,0672
	Ins/Ins=0,9343629344	Ins/Ins=0,9289617486	Ins/Ins=0,9312
	Del=0,03474903475	Del=0,03551912568	Del=0,0352
	Ins=0,9652509653	Ins=0,9644808743	Ins=0,9648

*Sampling power >80 indicates a good representation of the population (effect expressed in the population). Polymorphisms not associated by the meta-analysis (increased or reduced risk) did not have SP calculated; ** Frequency found using all study participants; *** Analyses conducted only with samples from countries arising from Spanish Colonization; **** Analyses conducted with Brazilian samples only, # Analysis unable to be completed due to lack of data.

Source: Author, 2023.
